# Binocular Diplopia: An Unusual Presentation of Squamous Cell Carcinoma of the Lung

**DOI:** 10.7759/cureus.27008

**Published:** 2022-07-19

**Authors:** Kyle Sugg, Waseem Diab, Aditi Kappagantu, Omid Yazdanpanah

**Affiliations:** 1 Department of Internal Medicine, Wayne State University Detroit Medical Center, Detroit, USA; 2 Department of Internal Medicine, Wayne State University School of Medicine, Detroit, USA

**Keywords:** squamous cell lung carcinoma, sellar metastasis, metastatic squamous cell carcinoma, squamous cell carcinoma of the lung, binocular diplopia

## Abstract

Here, we discuss the case of a 72-year-old male with a known history of COPD who presented with one month of binocular diplopia and headache. The initial clinical investigation discovered destructive intraosseous lesions within the sellar and para-sellar (SPS) regions, suggesting primary versus metastatic intracranial lesions. Further examination revealed a mass in the right lung, with subsequent biopsy confirming squamous cell carcinoma (SCC) of the lung as the primary site of malignancy. The SPS regions of the basicranium, while well-documented to be associated with various primary neoplasms, rarely serve as sites of metastasis. Throughout this article, we will review the pathophysiology of squamous cell lung cancer, current understandings of SPS metastasis, and considerations of metastatic lung SCC management.

## Introduction

Metastases involving the sellar and para-sellar (SPS) regions of the basicranium are notably rare; they represent less than 1% of all intracranial metastatic lesions and around 1% of pituitary masses that receive operative treatment [1,2]. While most cases of metastasis to the SPS region remain asymptomatic, those who develop symptoms typically do not do so until late in the course of disease progression. A predominance of these metastatic lesions involved the posterior aspect of the SPS region; this has been attributed to the posterior pituitary receiving blood flow from the systemic circulation, while the anterior pituitary receives its blood flow from the hypophyseal portal system [3]. A review of the medical literature demonstrates variable incidence of associated presenting symptoms that include visual disturbances, panhypopituitarism, diabetes insipidus, headache, cranial nerve palsy/ophthalmoplegia, and diplopia [1-5]. Primary malignancies most often associated with metastasis to the SPS region include breast and lung cancers, followed by renal, prostate, pancreatic, and colon cancers. Hematologic malignancies have also been reported, albeit they are much more uncommon [1,3].

Lung cancer has remained the leading cause of cancer mortality globally; approximately 85% of lung cancer cases involve the subtypes of non-small cell lung cancer (NSCLC), including squamous cell carcinoma (SCC) [6,7]. Most patients diagnosed with lung cancer will present with respiratory symptoms including cough, hemoptysis, dyspnea, and chest pain. Symptoms of distant metastasis, however, can be a less common initial presentation [8,9]. Among patients with NSCLC, brain metastasis is most frequent with adenocarcinoma and least frequent with squamous cell carcinoma. The risk of brain involvement is correlated with primary tumor size and the existence of regional node involvement [10]. This involvement of the central nervous system (CNS) can change the typical initial manifestations of lung cancer, instead leading to symptoms such as headache, vomiting, visual field loss, cranial nerve deficit, seizures, and hemiparesis. These atypical symptoms may delay diagnosis, as well as subsequent initiation of treatment.

## Case presentation

The patient was a 72-year-old male with a past medical history of hypertension, diabetes mellitus (type II), chronic kidney disease (stage IV), PTSD, COPD (GOLD stage C, not requiring home supplemental oxygen), and a 25 pack-year history of smoking tobacco (last cigarette approximately two years prior to arrival), who initially presented to the hospital following the onset of headache one month prior to arrival and diplopia two days prior to arrival. At the time of initial presentation, the headache was described as a constant “sharp” pain in the frontal scalp that did not radiate elsewhere, rated 8/10 in severity. This was not relieved with over-the-counter analgesics. The patient did endorse associated nausea, photosensitivity, and right-sided chest wall pain. He denied fever, fatigue, chills, dyspnea, cough, abdominal pain, nausea, vomiting, or any other acute medical complaints at that time. Subsequently, the patient was admitted for further evaluation of his headaches and pain control.

Presenting vital signs consistent with uncontrolled hypertension with a blood pressure of 175/108 mmHg, while all other vital signs were found to be within the normal range: temperature, 36.8°C; heart rate, 89 bpm; respiratory rate, 18 rpm; and oxygen saturation, 96% on room air. Physical examination demonstrated the presence of multiple cranial nerve palsy, sluggish reaction of the right pupil with restriction of adduction of the right eye leading to binocular diplopia (oculomotor nerve, CN III), and right eyelid ptosis with obliteration of the right nasolabial fold (facial nerve, CN VII). There were no visual field deficits, and the remainder of the neurologic examination was unremarkable. Examination of the lung fields showed that they were clear to auscultation bilaterally, and there was no evidence of conversational dyspnea. Following the administration of antihypertensive medications, blood pressure returned to the normotensive range.

**Figure 1:**
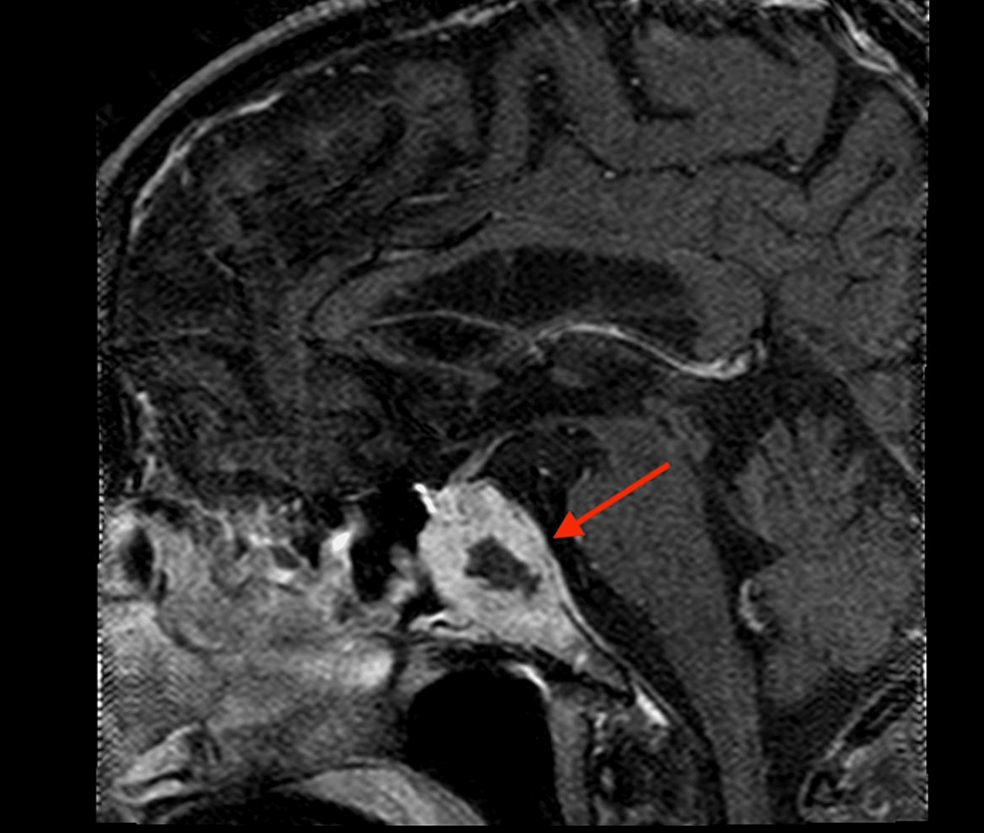
Sagittal MRI of the metastatic lesion abutting the optic chiasm (arrow).

**Figure 2:**
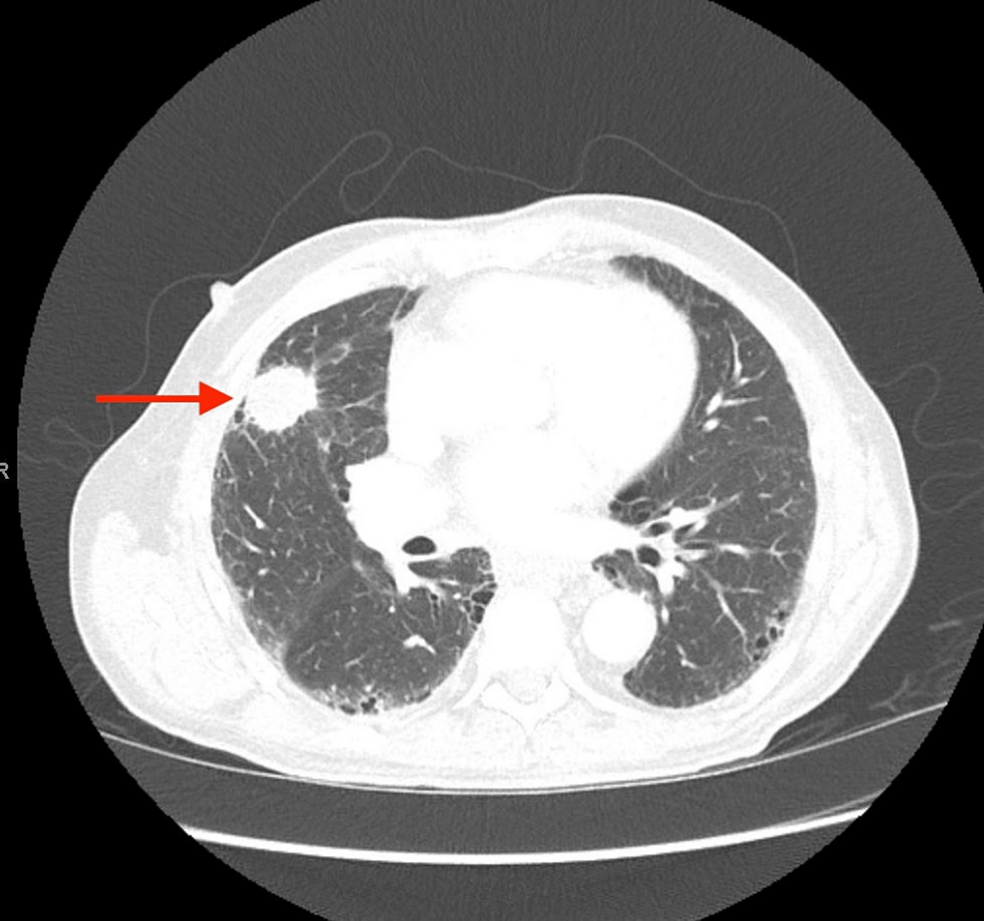
Axial chest CT demonstrating the presence of a right upper lobe spiculated mass (arrow).

**Figure 3:**
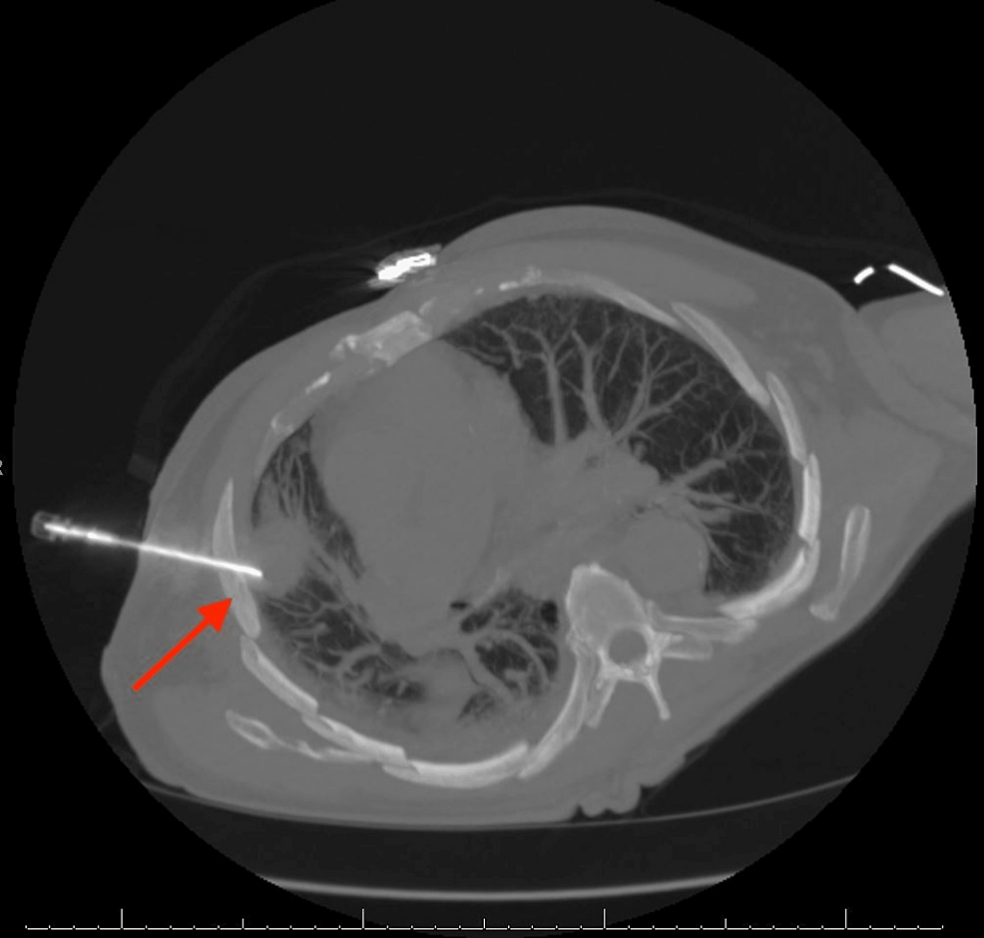
Core needle biopsy (CT-guided) of the right lung mass (arrow).

Imaging studies, including head CT and brain MRI, showed destructive intraosseous lesion within the basisphenoid with apparent involvement of the inferior recesses of the right cavernous sinus, the dorsum sella, and the floor of the sella turcica (Figure 1). Metastasis to the brain from a previously undiscovered primary malignancy was the most likely origin of this lesion; the tumor was near a highly vascular area, and primary brain malignancy is well known to be much more uncommon. Subsequent CT imaging of the chest/abdomen/pelvis with IV contrast discovered a large spiculated mass in the right upper lung lobe (Figure 2), without evidence of malignancy or metastatic disease in the abdomen or pelvis. Core biopsy results of this lung mass (Figure 3) were consistent with squamous cell carcinoma of the lung. Further investigations to identify other possible sites of primary malignancy were unremarkable, including a review of the most recent colonoscopy (performed within two years prior to hospitalization). Following meetings with Oncology and Palliative Care, the patient ultimately declined further treatment, entered hospice care, and passed away soon thereafter.

## Discussion

SCC of the lung, a subtype of NSCLC, represents the neoplastic transformation (and subsequent proliferation) of the squamous epithelial cells that line the airways of the lung. While SCC of the lung has a propensity to metastasize to certain regions of the body (e.g., liver, adrenal glands, and bone), it is noted to have limited distribution of metastasis in the brain attributed to a slow exophytic growth pattern [11,12]. The case presented above represents a notably rare site of lung SCC metastasis to the SPS regions of the basicranium.

A review of the medical literature demonstrates variable incidence of associated presenting symptoms when metastasis to the SPS regions of the basicranium is present. The manifesting symptoms most often result from increased intracranial pressure related to mass effect, as well as dysfunctional cerebrospinal fluid production and/or outflow. These include visual disturbances (reports vary from 30% to 62%), panhypopituitarism (37.7% to 59%), diabetes insipidus (27.4% to 50%), headache (20% to 47%), cranial nerve palsy/ophthalmoplegia (25% to 31%), and diplopia (17.4%) [1-5]. Patients may also present with increased mean arterial pressure, a physiologic response that assists in maintaining appropriate cerebral perfusion pressure.

In the case presented, the patient's presenting symptom was binocular diplopia. This refers to a double vision occurring when both eyes are open and absent when either eye is closed. This is the result of extraocular muscle dysfunction leading to poor alignment of the eyes, causing the individual to experience double vision. When one eye is closed, there are no competing visual inputs, subsequently resolving the diplopia. Demonstrating the association between our patient's metastatic lesions and the neuroanatomy surrounding them, the cranial nerves contained within the region of the sella turcica (CN III, IV, and VI) were affected in such a way to drive extraocular movement dysfunction.

There have been significant advancements in the medical treatment of advanced NSCLC in recent decades. However, these have largely been confined to patients with non-squamous histology. Due to a lack of comprehensive characterization of genomic alterations, even the most current molecular-targeted therapies for SCC of the lung have demonstrated poor efficacy. Current standard therapies meant to treat locally advanced or metastatic SCC of the lung typically involve two-drug chemotherapy regimens. These include either cisplatin or carboplatin, as well as a third-generation agent (e.g., gemcitabine, taxanes, or vinorelbine) [13].

Treatment choice becomes further complicated in the presence of metastatic lesions to the CNS, as drug delivery through the blood-brain barrier (BBB) becomes a key consideration [14,15]. The invasion of malignant cells places them within otherwise healthy brain tissue, in which the BBB effectively regulates the transport of these therapeutics. Although the BBB within the metastatic lesions may be weakened, the intact BBB of the surrounding healthy tissue provides the invading cells a level of protection from many systemically administered medications (thereby preventing them from reaching the intended target). Furthermore, brain tumors contain a higher cell density and collagen content than the surrounding brain tissue. These changes result in a heterogeneous pattern of BBB weakening, consequently leading to an unfavorable gradient for drug permeation. While novel drug delivery strategies are currently being investigated, CNS metastasis positions itself as a significant challenge to existing treatment strategies [15].

## Conclusions

Presented here is a case of metastatic lesion in the SPS regions of the basicranium, with SCC of the lung as the primary malignancy. Workup of SPS neoplasm must include a broad search for primary malignancy, if not already known. If SCC of the lung is found to be the source of primary malignancy, treatment regimens have relatively poor efficacy.
